# The Use of Structured Imagery and Dispositional Measurement to Assess Situational Use of Mindfulness Skills

**DOI:** 10.1371/journal.pone.0070253

**Published:** 2013-07-30

**Authors:** Jonathan C. Mitchell, Patricia A. Bach, Jeffrey E. Cassisi

**Affiliations:** Department of Psychology, University of Central Florida, Orlando, Florida, United States of America; Institute of Psychiatry at the Federal University of Rio de Janeiro, Brazil

## Abstract

The recent proliferation of studies on mindfulness produced varying theoretical models, each based in part on how mindfulness is assessed. These models agree, however, that mindfulness encompasses moment-to-moment or situational experiences. Incongruence between dispositional and situational assessment would be problematic for theory and empirical research. In particular, it remains to be established whether situational measurement is an accurate method for mindfulness assessment and whether dispositional measures are able to accurately detect mindfulness skills in various situations. The association between dispositional and situational mindfulness processes (i.e., situational attention awareness and emotion acceptance) was examined in two studies. In Study 1 (N = 148), independent groups who reported high and low levels of dispositional mindfulness skills were compared on a continuous measure of situational mindfulness skills. In Study 2 (N* = *317), dispositional mindfulness questionnaires were used to predict situational use of mindfulness skills. Results suggest not only that situational measures accurately detect use of mindfulness skills, but also that dispositional measures can predict one’s use of situational mindfulness skills. Findings from both studies were consistent across both positive and negative situations. Moreover, neither neuroticism nor extraversion was shown to have a moderating effect on the relationship between dispositional and situational use of mindfulness skills. The implications of these findings for clinical practice and future investigations pertaining to measurement validity in this area are discussed.

## General Introduction

Mindfulness has been defined variously, as a set of skills, as an ongoing process, and as an outcome of ongoing practice [1], [2]. In a comprehensive review of mindfulness theory, Bishop et al. [3] proposed that mindfulness encompasses present-moment attention awareness and acceptance of emotional, cognitive, and perceptual experiences. This basic operational definition is offered as a unifying model of the construct and is consistent with early descriptions of mindfulness in scientific literature [4]. Other models of mindfulness emphasize distinct theoretical facets (e.g., description, non-reactivity, non-distraction) and presume a dispositional approach to measurement [5], [6]. Indeed, a notable limitation of many empirical mindfulness studies is that the relevant skills are measured on a dispositional level alone [7], [8]. The primary objectives of the present investigation are to establish that situational measurement can accurately reflect dispositional measures of mindfulness skills, and to demonstrate that dispositional measures of mindfulness skills generalize to a range of situations regardless of personality traits.

Self-report mindfulness questionnaires have been central to many investigations pertaining to both theory and intervention development. These questionnaires have not only increased in popularity but also withstood psychometric scrutiny. Two prominent examples of such measures are Baer, et al.’s [5] Kentucky Inventory of Mindfulness Skills (KIMS) and Hayes et al.’s [9] Acceptance and Action Questionnaire (AAQ). The KIMS separates mindfulness into component processes based on a clinical intervention [10]. This questionnaire comprises four factors (observing, describing, acting with awareness, and accepting without judgment) that represent separate but related skills. The AAQ was developed to measure an individual’s tendency toward experiential avoidance: an unwillingness to remain in contact with negatively evaluated private events, and subsequent behaviors intended to alter the form or frequency of these experiences [5], [6]. Experiential avoidance is considered the antithesis of mindful acceptance. There is support for the reliability and validity of these questionnaires [8], [11]. However, the degree to which they generalize to situational processes has not been as thoroughly investigated. In keeping with this notion, mindfulness measurement should occur at both the dispositional and situational level. Incongruence between dispositional and situational assessment would be problematic for theory and empirical research.

Several researchers have examined mindfulness skills as they relate to moment-by-moment behavior. These investigations predominantly address behavior related to various clinical symptoms such as panic response [12], [13] and intrusive thoughts [14]. These investigations each seem to agree that use of mindfulness skills reduces the distress associated with these symptoms. While these investigations measure situational behavioral responses, they are limited in that they do not measure core mindfulness skills at the time they are utilized. Moreover, these studies focus solely on measuring responses to aversive stimuli. A promising method of assessing situational mindfulness skills is structured imagery. This method provides an opportunity to probe both situational attention awareness, and emotion acceptance in a greater variety of positive and negative situations than is possible with *in vivo* approaches.

To date, one previous investigation has used imagery to study mindfulness. Heeren, van Broeck, and Philippot [15] attempted to clarify the effect of mindfulness on autobiographical memory. The authors asked two groups of participants (mindfulness training group and control group) to respond to emotional cue words such as *lucky* and *guilty,* by cultivating memories related to those prompts. As predicted, assessments pre- and post-intervention indicated that mindfulness training increased specific memory recall and decreased general memory recall, outcomes that are closely associated with automatic attentional deployment. This investigation lends support to the methodology developed in the present investigation, and also suggests that continued evaluation of situational mindfulness processes can inform theory development and further empirical research.

One final consideration pertinent to the present investigation involves the impact of individual differences on the relationship between dispositional and situational behavior. Some research suggests that dispositional personality traits and evaluative processing tendencies interact to influence situational emotional and behavioral responses [16], [17]. These relationships need to be understood in the application of situational measures of mindfulness. Indeed, Robinson et al. [16] caution against assuming that the influence of individual differences (i.e. evaluation and personality) remains consistent across various situations. Therefore, it is important to determine whether personality traits moderate the relationship between the dispositional and situational use of mindfulness skills.

In light of these distinct, yet related issues, this investigation is based on two independent studies. Study 1 aims to demonstrate that the situational measurement of mindfulness skills reflects the same behaviors as current dispositional measures. Study 2 aims to demonstrate that individual’s self-reported dispositional use of mindfulness skills can predict situational use of mindfulness skills in both positive and negative situations, regardless of personality traits.

### Study 1 Introduction

Mindfulness training has become increasingly popular in clinical practice, and its accurate measurement and assessment are important. It has been documented that current self-report questionnaires oriented toward dispositional use of mindfulness skills are able to detect between- and within-individual differences [18], [19], [20]. Several situational mindfulness inventories have been created (e.g., Freiberg Mindfulness Inventory, [21]; Toronto Mindfulness Scale, [22]); however, these inventories were designed specifically to assess mindful states following formal meditation practice. It is unclear whether situational mindfulness can be accurately assessed across a range of experiences. In particular, a primary question that remains to be answered is whether measures of situational mindfulness skills provide an accurate assessment of the same underlying construct as dispositional measures. Therefore, following Bishop et al.’s [3] operational definition of mindfulness, we hypothesize that situational measurement of mindfulness will reveal that those with a greater tendency toward dispositional use of mindfulness skills will also report greater tendency toward situational use of mindfulness skills.

### Study 2 Introduction

Extant literature has demonstrated the utility of dispositional measurement of mindfulness skills in a number of clinical settings [12], [14], [28]. While the findings from Study 1 offer partial support to the validity of situational measurement, it is important to also examine whether dispositional measures reliably predict individual’s situational use of mindfulness skills both positive and negative situations. One crucial consideration, however, involves the extent to which personality traits affect one’s use of mindfulness skills.

The relationships among dispositional personality traits and situational behavioral tendencies have been closely examined in previous research. In particular, individuals high in extraversion are known to disproportionality attend to and appraise appetitive stimuli positively, whereas individuals high in neuroticism disproportionality attend to and appraise neutral stimuli negatively [29], [30], [31]. Most mindfulness inventories are not correlated with extraversion, although extraversion has been shown to be negatively correlated with experiential avoidance [6]. However, neuroticism, has been found to be negatively correlated with measures of mindfulness [5] and positively correlated with measures of experiential avoidance [9]. These findings raise the possibility that neuroticism and extraversion may differentially impact situational mindfulness. Therefore, it is crucial to examine whether these personality traits affect the strength of the relationship between dispositional and situational use of mindfulness skills in both positive and negative situations.

It is hypothesized therefore that high levels of dispositional attention awareness will predict greater attention awareness in imagined situations. We further hypothesize that low levels of dispositional acceptance and high levels of dispositional experiential avoidance will predict low levels of emotion acceptance in imagined situations. We expect that these patterns of responses will remain consistent across positive and negative situations. Finally, regardless of situational valence, it is hypothesized that the relationships between dispositional mindfulness skills and situational mindfulness skills will remain significant even after accounting for the effect of personality traits.

## Methods

All participants in studies 1 and 2 provided informed consent electronically and were assigned a random identification number to insure confidentiality. The University of Central Florida institutional review board approved the participant consent procedure as well as all other study procedures.

### Study 1 Participants

A sample of 442 undergraduate students at a large southeastern university participated in this investigation for course credit. Of these, respondents who fell within the bottom quartile of completion time (30 minutes or less), who endorsed any of the validity scale items described below, or who were identified as multivariate outliers based on Mahalanobis distance values (*p*<.001) were excluded (N = 125) prior to analysis [23]. Participants who fell within the top and bottom decile along the dimensions of dispositional attention awareness (KIMS Observe subscale score) and dispositional emotion acceptance (KIMS Accept without Judgment subscale score) were then placed into four distinct dispositional groups: high (N = 38; 27 females; *M*_age_ = 19.34, *SD* = 2.73) and low (N = 38; 23 females; *M*_age_ = 19.16, *SD* = 1.42) dispositional attention awareness, as well as high (N = 40; 31 females; *M*_age_ = 19.29, *SD* = 1.38) and low (N = 32; 26 females; *M*_age_ = 19.00, *SD* = 1.15) dispositional emotion acceptance groups. Differences in group size are attributable to slight skewness in the dependent variable. These subscales were selected to identify high and low dispositional groups based on the similarity between their content and the theoretical description of core mindfulness components offered by Bishop et al. [3]. The decision to create high and low dispositional groups was based on previous research that utilized a similar approach in order to identify how behavioral responses differ among those at extreme ends of a continuous dimension [13], [24]. Although the decision to utilize these two scales was made *a priori*, correlation analyses were examined *post hoc* in order to determine whether other subscales would provide additional information in the between-group analyses. These findings demonstrated that the KO and KAWJ demonstrated a more consistent pattern of association with the situational dependent variables (see Table 1). Therefore, these scales were chosen in the interest of maintaining parallel form in all analyses.

**Table 1:** Zero-Order Correlations, Means, Standard Errors, Standard Deviations, and Ranges of Study Variables.

Scale	1	2	3	4	5	6	7	8	9	10	11
1. Neuroticism	–										
2. Extraversion	−.36***	–									
3. AAQ	.58***	−.31***	–								
4. KO	.12*	.11*	.00	–							
5. KD	−.21***	.29***	−.29***	.25***	–						
6. KAWA	−.29***	.07	−.39***	−.02	.19**	–					
7. KAWJ	−.48***	.22***	−.51***	−.18**	.22***	.27***	–				
8. SAA-Positive	−.06	.10	−.12*	.19**	.15**	.14**	.06	–			
9. SEA−Positive	−.15**	.13*	−.23***	.04	.11*	.06	.21***	.50***	–		
10. SAA−Negative	−.04	.10	−.04	.17**	.13*	.06	.04	.86***	.50***	–	
11. SEA−Negative	−.17**	.15**	−.23***	−.05	.06	.14**	.26***	.19**	.57***	.20***	–
Statistic											
Mean	5.97	7.72	33.63	41.27	26.01	28.54	30.26	157.29	45.45	155.88	20.91
SE of the Mean	0.19	0.21	0.38	0.43	0.37	0.43	0.43	1.71	1.33	1.75	1.36
SD	3.30	3.82	6.65	7.72	6.52	5.74	7.66	30.51	23.68	31.20	24.28
Range	0−12	0−12	15−40	21−39	11−29	13−33	9−36	75−200	−22−75	61−200	−54−75

Note: AAQ = Acceptance and Action Questionnaire; KIMS = Kentucky Inventory of Mindfulness Skills; KO = KIMA Observe subscale; KD = KIMS Describe subscale; KAWA = KIMS Act with Awareness subscale; KAWJ = KIMS Accept without Judgment subscale; SAA = Situational Attention Awareness; SEA = Situational Emotion Acceptance

* *p*<0.05,

** *p*<0.01,

*** *p*<0.001.

### Study 2 Participants

The sample in study 2 included 317 participants (230 female) who ranged in age from 18 to 32 (*M = *19.23, *SD = *2.26). This sample was distinct from the sample in Study 1, and was subject to the same data reduction procedure described above. The majority of participants identified as Caucasian (70.3%), with smaller proportions of participants identifying as Hispanic (11.7%), Asian/Pacific Islander (7.3%), Black (5.7%) and Multiracial (5.0%). The majority of participants reported their marital status as single (97.5%) and reported no history of psychotherapy (95.3%). All participants provided informed consent electronically and were assigned a random identification number to insure confidentiality. The University of Central Florida institutional review board approved the participant consent procedure as well as all other study procedures.

### Study 1 Measures and Procedure

#### Demographic survey

Participants completed an eight-item demographic questionnaire. Questions in this survey addressed age, gender, ethnicity, marital status, academic year, average yearly household income, and personal history of mental health or psychopharmacological treatment.

#### Millon Clinical Multiaxial Inventory-III-V Scale (MCMI-III-V; [25])

To ensure response validity within the self-report measures, the validity scale (V) items of the MCMI-III were inserted randomly into the item pool. The V scale contains three true/false items, each representing an exceptionally peculiar statement (e.g. “I have not seen a car in the last ten years”). Endorsement of any of these items indicates a questionable response set.

#### Kentucky Inventory of Mindfulness Skills (KIMS; [5])

The KIMS is a 39-item self-report mindfulness measure comprising four subscales: Observe (KO), which measures the ability to notice and attend to the details of present-moment stimuli, Describe (KD), which measures the ability to briefly and accurately label internal and external stimuli, Act with Awareness (KAWA), which measures the ability to focus on internal and external experiences without distraction, and Accept without Judgment (KAWJ), which measures the ability to allow experiences of the present moment to occur without evaluating them. Higher subscale scores indicate higher levels of each facet of mindfulness. Participants respond to a 5-point Likert scale (1 = *never true*, 5 = *always true*) indicating how often they experience the internal events presented in each item. The authors report very good to excellent internal consistency for all subscales (α’s = .76–.91.). In the present study, a similar level of internal consistency on these subscales was observed (α’s = .76–.89.).

#### Situational assessment of mindfulness skills

Participants were presented with descriptions of ten everyday situations to prompt emotional responses, as well as associated self-report items were administered to assess situational mindfulness processes. These brief, narrative scenarios were based on the Measure of Awareness and Coping in Autobiographical Memory (MACAM; [26]). The original instrument contains a series of written vignettes accompanied by brief self-report ratings. The original scenarios included positively and negatively valenced language intended to induce positive and negative emotional responses, respectively. Ten scenarios were selected based on their relevance to the undergraduate population and the frequency with which this population would likely experience the scenarios. The research team then modified the language in the scenarios by removing statements describing what emotions should be experienced (e.g., “you now feel sad”). This was done to avoid biasing emotional responses and to allow participants to respond naturally, as they typically would in that context.

The 10 scenarios used in the present investigation were presented via separate audio recordings. Five of the scenarios described pleasant everyday social situations (e.g., you and a friend make plans for a relaxing summer vacation) and five described unpleasant everyday social situations (e.g., you are stood up on a date and later find the individual spending time with someone else). Before the scenarios were presented, participants were encouraged to engage in the imaginal portion in a quiet environment. Each recording was approximately one minute in length, and the order of scenario presentation was randomized. Recorded instructions directed participants to “picture yourself in this situation for 15–30 seconds.”

After listening to each scenario, participants immediately responded to a total of 13 questions regarding their experience during the imagery. First, a single clarity item (“Please rate how clearly you were able to imagine yourself in the situation”), based on 10-point Likert scale (1 = *not at all*, 10 = *very much so*) was included to assess participant’s immersion during imagery. Subsequently, participants rated the extent to which they were *attentive*, *focused* and *engaged* in the experience. These three items were constructed to evaluate the participants’ levels of situational attention awareness and were also based on a 10-point Likert scale. A composite score labeled *Situational Attention Awareness* (SAA) was derived from the unweighted sum of participants’ ratings on these three items in both the positive and negative structured vignettes. High scores on this measure indicate greater level of attention awareness. Internal consistency on this measure was excellent (α = 0.96).

Next, participants rated the degree to which they experienced six emotions (happy, angry, sad, scared, disgusted, and surprised) during the imagery. These ratings were obtained to ensure the imagery protocol was effective. Emotions were chosen based on Ekman et al.’s [27] work identifying core human emotions. Ratings for each emotion were made on a 7-point Likert scale (1 = *not at all*, 7 = *very much so*) and were summed in both the positive and negative scenarios. Internal consistency on these ratings ranged from adequate to good (α = .70–.82).

Finally, participants rated their assessment of experienced emotions during each scenario on three separate 10-point Likert scales. Prompted by the phrase “My emotional reactions to this scenario were,” participants rated whether their emotional experience was *good* or *bad*, *appropriate* or *inappropriate*, and *proper* or *improper*. These ratings were intended to capture the degree to which participants’ appraised their emotions and responded with judgment or evaluation (i.e., emotion non-acceptance). An emotional response acceptance composite score labeled *Situational Emotion Acceptance* (SEA) was derived from the unweighted sum of participants’ ratings on the three items in both the positive and negative structured vignettes. High scores on this measure indicates greater emotion acceptance. Internal consistency on this measure was excellent (α = 0.94). All scenarios and self-report items are available from the first author upon request.

Data were collected electronically through a university-based research participation website. At the beginning of the study, participants read a brief description of the study, provided their informed consent, and were assigned a random identification number to insure confidentiality. Instructions for self-report questionnaires accompanied the corresponding items in the same manner indicated in the original documentation and were presented in the same order for all participants. Participants were directed to an outside webpage to listen to the audio recordings, imagine themselves in the vignettes, and respond to the SAA and SEA items. At the conclusion of the study, participants were debriefed and provided contact information for the research team as well as the university institutional review board.

### Study 2 Materials and Procedure

The data collection procedure was identical to that described in Study 1. Materials for study two included the same self-report measures described in Study 1 as well as two additional self-report questionnaires, described below.

#### Eysenck Personality Questionnaire – Revised Short Scale (EPQ-RS; [32])

The EPQ-RS consists of 57 items that measure dispositional levels of extraversion, neuroticism, and psychoticism. Respondents are asked to indicate whether a particular statement applies to them on a forced choice (yes/no) basis. The subscales of the EPQ-RS have demonstrated good to very good internal consistency (Cronbach’s α = 0.66 to 0.86). In the present study, the Cronbach’s alphas for the Neuroticism and Extraversion scales were .81 and .84, respectively. The construct of psychoticism is not theoretically related to mindfulness, and therefore was not included in the analyses.

#### Acceptance and Action Questionnaire (AAQ; 9)

The AAQ is a 9-item instrument that assesses dispositional levels of experiential avoidance. Respondents are asked to indicate the extent to which each statement is true for them on a 7-point Likert scale (1 = *never true*, 7 = *always true*). The AAQ yields a single composite score, with higher scores indicating higher levels of experiential avoidance. The AAQ has demonstrated good internal consistency (α = .70). Internal consistency for the AAQ in this study was adequate (α = .65).

## Results and Discussion

### Study 1

Descriptive statistics were calculated to verify the equivalence of participant characteristics in each of the groups. First, high and low dispositional groups were compared across demographic variables. The high and low dispositional attention awareness and emotion acceptance groups did not differ from one another with regard to age (*p*s>.76) or qualitatively with regard to gender, ethnicity, marital status, academic year, average household income, and other historical variables (*p*s>.14). Because the SAA and SEA variables were scaled differently, these variables were standardized (*z*-score transformed) prior to data analysis for ease of interpretation.

All vignettes were subjected to response manipulation checks. Paired samples *t*-tests were conducted on each of the emotion ratings in the positive and negative scenarios to ensure structured imagery elicited intended emotional reactions. As expected, within the final sample, positively valenced scenarios were associated with significantly higher ratings of happiness, *t*(316)* = *47.02, *p*<.001, η^2^ = 0.30, while negatively valenced scenarios were associated with significantly higher ratings of anger, *t*(316) = −36.77, *p*<.001, η^2^ = 0.13, sadness, *t*(316) = −32.17, *p*<.001, η^2^ = 0.09, fear, *t*(316) = −8.22, *p*<.001, η^2^ = 0.43, disgust, *t*(316) = −26.22, *p*<.001, η^2^ = 0.11, and surprise, *t*(316) = −7.22, *p*<.001, η^2^ = 0.20. Responses to the single clarity item between the positive and negative scenarios were also compared. As expected, clarity ratings for the positive (*M* = 41.31, *SD = *7.70) and negative scenarios (*M* = 40.89, *SD* = 8.32) were not significantly different, *t*(316) = 1.65, *p>*.10.

Subsequently, separate 2 (Dispositional Group: low vs. high) X 2 (Situation Valence: positive vs. negative) mixed model multivariate analysis of variance (MANOVAs) were calculated to examine differences in situational mindfulness skills in both pairs of dispositional groups. Analyses presented here utilized the criterion of Wilks’ Lambda (Λ) to test main effects and interactions. Planned comparisons were used to test between-group differences.

**Figure 1:**
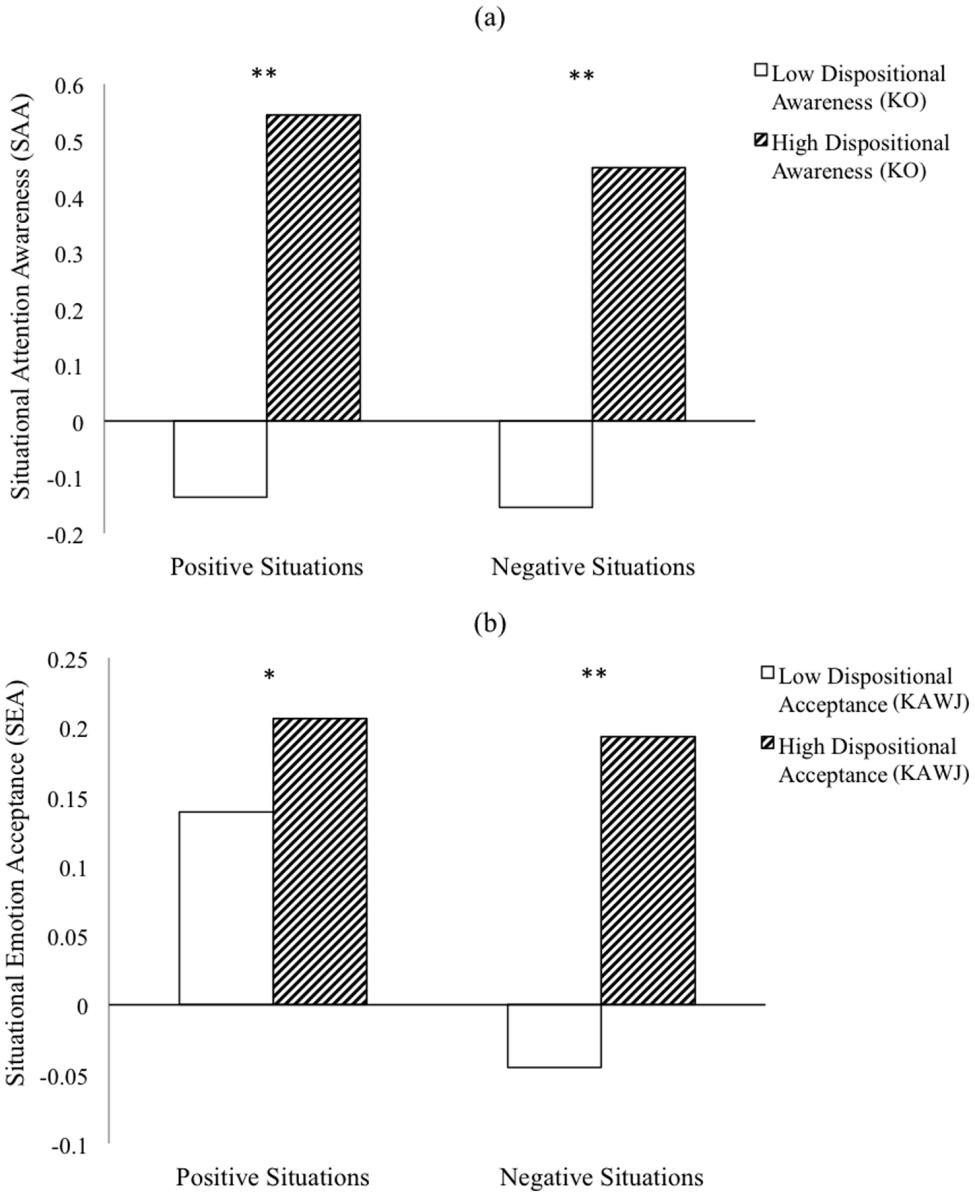
Z-score Transformed Situational Attention Awareness Scores (a) and Situational Emotion Acceptance Scores (b) Among High and Low Dispositional Groups. *p<.05. **p<.01.

With regard to the dispositional awareness groups, multivariate results of the omnibus test were significant, Λ = .88, *F*(2,73) = 4.92, *p* = .01, η^2^ = 0.12, and revealed main effects for dispositional group in both the positive, *F*(1,74) = 10.00, *p* = .002, η^2^ = 0.13, and negative situations, *F*(1,74) = 7.20, *p*<.001, η^2^ = 0.09. Planned comparisons confirmed that individuals higher in dispositional attention awareness also responded with increased situational attention awareness at each valence level (*p*s <.01), There was no main effect for valence, (*F* <1), nor was there a group-by-valence interaction (*F* <1). A similar trend was observed with regard to the dispositional emotion acceptance groups. Multivariate omnibus results were significant, Λ = .98, *F*(2,69) = 4.61, *p* = .01, η^2^ = 0.12, and revealed main effects for group in both the positive, *F*(1,70) = 5.06, *p* = .028, η^2^ = 0.07, and negative situations, *F*(1,70) = 8.93, *p* = .004, η^2^ = 0.11. Planned comparisons confirmed that individuals higher in dispositional attention awareness also responded with increased situational attention awareness at each valence level (*p*s <.01). There was no main effect for valence (*F* <1). All between-group differences observed in this analysis are depicted visually in Figure 1.

In support of the first hypothesis, individuals who reported high dispositional use of mindfulness skills also evidenced high situational use of mindfulness skills across a range of emotionally evocative scenarios. Specifically, when a sample of participants was dichotomized based on observed levels of dispositional mindful attention and acceptance, individuals with the highest levels of these traits responded with similar situational ratings when asked to imagine themselves in a variety of both positive and negative vignettes.

Within measures of situational attention awareness, it is interesting to note that the low and high dispositional groups differed by a similar order of magnitude in both positive and negative vignettes. That is, regardless of the emotional valence of the situation, both high and low acceptance groups evidenced an analogous discrepancy in their respective mean ratings. This pattern of findings suggests relative stability in the dispositional and situational use of mindful attention. The difference between ratings of situational emotion acceptance in positive and negative situations, however, was much less stable. While the high and low dispositional acceptance groups reported significantly different ratings of situational emotion acceptance, the relative difference between both groups’ ratings in both positive and negative situations was inconsistent. That is, those in the low dispositional acceptance group reporting lower SEA scores than the high dispositional group in the negative, as opposed to the positive vignettes. This pattern would suggest that while emotional valence of a given situation may affect an individual’s level of acceptance, the level of attention awareness is less prone to fluctuation.

These results are intriguing; however, because they are based on an artificial dichotomization of dispositional skills, they may obscure the dispositional-situational relationship across the full spectrum of attentional and acceptance domains. Further analyses utilizing the range of attentional and acceptance experiences can offer additional support for the relationship between dispositional and situational measures. In addition, the extent to which individual differences (i.e., personality traits) may alter the relationship between dispositional and situational use of mindfulness skills remains unclear. In study 2, these issues are explored in greater depth in an effort to clarify the strength of the relationship between dispositional and situational use of mindfulness skills.

### Study 2

Previous research indicates that gender interacts with a number of dispositional personality traits [16], [33]. In consideration of these findings, an important initial task was determining whether participant gender was uniquely associated with either independent or dependent variables. Independent samples *t*-tests revealed that males and females did not differ significantly on measures of dispositional attention awareness, *t*(315) =  −.78, *ns*, or emotion acceptance, *t*(315) =  −.21, *ns*, but did differ on neuroticism, *t*(315) = −3.64, *p*<.01. Participant age and ethnicity were also not related to indicator variables (all *p*s *>*.75) or dependent variables (all *p*s >.21) and were therefore not included in the final moderation analyses. Zero-order correlations among study variables as well as means, standard deviations and ranges of all self-report measures are presented in Table 1.

#### Moderation analyses

Moderation analyses were conducted through a series of hierarchical linear regression models in accordance with best practice guidelines [34]. A moderation approach was used because extraversion and neurotcism are theoretically more likely to act as moderators than as mediators; that is, they are more likely to alter the strength of the relationship between dispositional and situational variables than they are to explain the dispositional-situational relationship itself. Prior to the analyses, variables were centered in order to minimize multicolinearity [35].

All moderation models were executed in the same basic two-step format. Step one included the independent variable (i.e., dispositional attention attention awareness, dispositional emotion acceptance, or experiential avoidance) and moderator variable (i.e., extraversion or neuroticism) specific to that model. Step two included an interaction term between each independent variable and it’s corresponding moderator variable. Variables were entered into the model in forced entry fashion. Neuroticism and extraversion moderation models were run seperately for each dependent variable (i.e., situational attention attention awareness, and situational emotion acceptance) across both valence categories (i.e, positive and negative vignettes), yielding 8 distinct mediation models. Four additional moderation models examining SEA were tested using the AAQ as an independent variable. For ease of interpretation, only standardized regression coeficients, analysis of variance (ANOVA) *F* ratios, and change in *F* ratios between model steps are reported in-text.

#### Models of Situational Attention Awareness (SAA)

Moderation analyses examining SAA included the KIMS Observe (KO) subscale as the primary independent variable. With regard to the moderating effects of neuroticism (N), findings from step one in the models are that KO was the only variable significantly related to SAA (β = .18–.20, *p*s <.01) in both the positive, *F*(2, 314) = 7.12, *p* = .001, and negative vignettes, *F*(2, 314) = 5.22, *p* = .006. When the KO x N interaction term was added in step two, the resulting model did not improve, Δ*F* <.90, *ns*, and the relationship between KO and SAA remained significant, β = .17–.19, *p*s <.01. A similar pattern was observed in moderation analyses including extraversion (E). Findings from step one confirmed that KO was significantly related to SAA (β = .17–.18, *p*s<.01) in both the positive, *F*(2, 314) = 6.93, *p* = .001, and negative vignettes, *F*(2, 314) = 5.51, *p* = .004. When the KO x E interaction term was added in step two, the resulting model did not improve, Δ*F*<2.00, *ns*, and the relationship between KO and SAA remained significant, β = .17, *p*s <.01. Model summaries for these analyses are presented in Table 2.

**Table 2:** Unstandardized Betas (Standard Error), 95% Confidence Intervals, Standardized Betas and Change in R^2^ Values for Neuroticism (Model A) and Extraversion (Model B) Moderation Analyses of Situational Attention Awareness in Positive and Negative Situations.

	Positive Situations				Negative Situations			
	B (SE)	95% CI	β	Δ*R* ^2^	B (SE)	95% CI	β	Δ*R* ^2^
**Model A**								
*Step 1*				.044				.032
KO	0.79 (.22)	0.36–1.23	.20***		0.71 (.23)	0.26–1.15	.18**	
N	−0.80 (.51)	−1.81–0.21	−.09		−0.64 (.53)	−1.68–0.39	−.07	
*Step 2*				.003				.002
KO	0.76 (.22)	0.33–1.20	.19***		0.68 (.23)	0.23–1.13	.17**	
N	−0.80 (.51)	−1.81–0.21	−.09		−0.64 (.53)	−1.67–0.04	−.07	
KO×N	−0.08 (.06)	−0.18–0.10	−.05		−0.05 (.06)	−0.18–0.07	−.05	
**Model B**								
*Step 1*				.042				.034
KO	0.72 (.22)	0.29–1.15	.18***		0.64 (.23)	0.19–1.08	.16**	
E	0.62 (.44)	−0.26–1.49	.08		0.65 (.46)	−0.25–1.55	.08	
*Step 2*				.000				.006
KO	0.72 (.22)	0.28–1.16	.18***		0.68 (.23)	0.23–1.13	.17**	
E	0.62 (.45)	−0.26–1.50	.08		0.57 (.46)	−0.33–1.47	.07	
KO×E	−0.01 (.06)	−0.11–0.11	.01		−0.08 (.06)	−0.20–0.03	−.08	

Note: KO = KIMS Observe subscale; N = Neuroticism; E = Extraversion;

** *p*<0.01,

*** *p*<0.001.

Overall, neither neuroticism nor extraversion moderated the relationship between dispositional and situational attention awareness. This finding is further supported by consistent regression coefficients across both positive and negative scenarios, demonstrating the stability of the dispositional-situational relationship in the domain of mindful attention.

#### Models of Situational Emotion Acceptance (SEA)

Moderation analyses examining SEA included the KIMS Accept without Judgment (KAWJ) subscale as the primary independent variable. With regard to the moderating effects of neuroticism, findings from step one in the models are that KAWJ was the only variable significantly related to SEA (β = .18–.23, *p*s <.01) in both the positive, *F*(2, 314) = 7.80, *p*<.001, and negative vignettes, *F*(2, 314) = 11.48, *p*<.001. When the KAWJ x N interaction term was added in step two, the resulting model did not improve, Δ*F* <0.66, *ns,* and the relationship between KAWJ and SEA remained significant, β = .19–.22, *p*<.01. A similar pattern was observed in extraversion moderation analyses. Findings from step one confirmed that KAWJ was significantly related to SEA (β = .19–.24, *p*s<.01) in both the positive, *F*(2, 314) = 8.43, *p*<.001, and negative vignettes, *F*(2, 314) = 12.84, *p*<.001. When the KAWJ x E interaction term was added in step two, the resulting model did not improve, Δ*F*<1.24, *ns*, and the relationship between KAWJ and SEA remained significant, β = .19–.24, *p*<.01. Model summaries for these analyses are presented in Table 3.

**Table 3:** Unstandardized Betas (Standard Error), 95% Confidence Intervals, Standardized Betas and Change in R^2^ Values for Neuroticism (Model A) and Extraversion (Model B) Moderation Analyses of Situational Emotion Acceptance in Positive and Negative Situations.

	Positive Situations				Negative Situations			
	B (SE)	95% CI	β	Δ*R* ^2^	B (SE)	95% CI	β	Δ*R* ^2^
**Model A**								
*Step 1*				.052				.072
KAWJ	0.55 (.20)	0.16–0.93	.18**		0.72 (.20)	0.33–1.11	.23***	
N	−0.47 (.45)	−1.35–0.48	−.07		−0.44 (.46)	−1.33–0.46	−.06	
*Step 2*				.002				.001
KAWJ	0.58 (.20)	0.19–0.98	.19**		0.70 (.20)	0.30–1.10	.22***	
N	−0.45 (.45)	−1.35–0.42	−.06		−0.45 (.46)	−1.34–0.46	−.06	
KAWJ×N	−0.04 (.05)	−0.15–0.06	−.04		0.03 (.05)	−0.08–0.13	.03	
**Model B**								
*Step 1*				.051				.074
KAWJ	0.60 (.17)	0.25–0.93	.19**		0.75 (.18)	0.40–1.09	.24***	
E	0.53 (.35)	−0.16–1.22	.09		0.59 (.36)	−0.11–1.29	.09	
*Step 2*				.002				.000
KAWJ	0.57 (.17)	0.23–0.92	.19**		0.75 (.17)	0.40–1.10	.24***	
E	0.58 (.35)	−0.12–1.27	.09		0.58 (.36)	−0.13–1.28	.09	
KAWJ×E	0.05 (.04)	−0.04–0.14	.06		−0.02 (.05)	−0.11–0.08	−.02	

Note: KAWJ = KIMS Accept without Judgment subscale; N = Neuroticism; E = Extraversion;

** *p*<0.01,

*** *p*<0.001.

Overall, neither neuroticism nor extraversion moderated the relationship between dispositional and situational emotion acceptance. This finding is further supported by the consistency across both positive and negative scenarios, which demonstrates stability of the dispositional-situational relationship in the domain of mindful acceptance.

In order to provide convergent support for these results, the same relationships were modeled using the AAQ as an indicator variable to predict situational emotion acceptance. In theory, this model should yield similar results as the models utilizing the KAWJ subscale, but in the opposing direction. With regard to the moderating effects of neuroticism, findings from step one in the models were that AAQ was the only variable significantly related to SEA (β = −.20– −.22, *p*s <.01) in both the positive, *F*(2, 314) = 8.98, *p*<.001, and negative vignettes, *F*(2, 314)* = 9.40, p*<.001. When the AAQ x N interaction term was added in step two, the resulting model did not improve, Δ*F* <0.14, *ns,* and the relationship between AAQ and SEA remained significant, β = −.21– −.22, *p*s <.01. A similar pattern was observed in extraversion moderation analyses. Findings from step one confirmed that AAQ was significantly related to SEA (β = −.21, *p*s <.01) in both the positive, *F*(2, 314) = 9.53 *p*<.001, and negative vignettes, *F*(2, 314) = 10.16, *p*<.001. When the AAQ x E interaction term was added in step two, the resulting model did not improve, Δ*F* <2.39, *ns,* and the relationship between AAQ and SEA remained significant, β = −.21, *p*s <.001. Model summaries for these analyses are presented in Table 4.

**Table 4:** Unstandardized Betas (Standard Error), 95% Confidence Intervals, Standardized Betas and Change in R^2^ Values for Neuroticism (Model A) and Extraversion (Model B) Moderation Analyses of Situational Emotion Acceptance Using the AAQ as an Indicator.

	Positive Situations				Negative Situations			
	B (SE)	95% CI	β	Δ*R* ^2^	B (SE)	95% CI	β	Δ*R* ^2^
**Model A**								
*Step 1*				.052				.056
AAQ	−0.76 (.24)	−1.22– −0.93	−.22***		−0.73 (.24)	−1.21– −0.26	−.20**	
N	−0.19 (.48)	−1.13–0.75	−.03		−0.38 (.49)	−1.34–0.59		
*Step 2*				.007				.006
AAQ	−0.76 (.26)	−1.22– −0.30	−.22***		−0.74 (.24)	−1.21– −0.26	−.21**	
N	−0.19 (.48)	−1.14–0.75	−.03		−0.38 (.49)	−1.34–0.59		
AAQ×N	0.08 (.05)	−0.03–0.19	.08		0.08 (.05)	−0.03–0.20		
**Model B**								
*Step 1*				.057				.061
AAQ	−0.74 (.20)	−1.14– −0.35	−.21***		−0.75 (.21)	−1.16– −0.34	−.21***	
E	0.39 (.36)	−0.31–1.09	.06		0.52 (.37)	−0.20–1.24	.08	
*Step 2*				.002				.007
AAQ	−0.76 (.20)	−1.14– −0.35	−.21***		−0.75 (.21)	−1.15– −0.34	.21***	
E	0.45 (.36)	−0.26–1.17	.07		0.63 (.37)	−0.10–1.36	.10	
AAQ×E	−0.04 (.05)	−0.14–0.05	−.05		−0.08 (.05)	−0.17–0.20	−.08	

Note: AAQ = Acceptance and Action Questionnaire; N = Neuroticism; E = Extraversion;

** *p*<0.01,

*** *p*<0.001.

These experimental models explained only a relatively small proportion of the variance in the situational attention awareness and emotion acceptance (*R* ^2^ = .03–.07). However, these values were not significantly altered when accounting for the moderating effects of the personality variables, providing further evidence for the stability of the relationship between dispositional and situational use of mindfulness skills. Moreover, these values are minimally problematic given the established limitations of the *R* ^2^ metric in measuring situational behavior (described below) as well as the primary aim of demonstrating a relationship between dispositional and situational measurement.

In support for the second hypothesis, higher levels of dispositional use of mindfulness skills predicted greater situational use of the corresponding skills. In particular, the KO scale was a significant predictor of situational attention awareness, and both the AAQ and KAWJ scale were significant predictors of situational emotion acceptance. Furthermore, this pattern was consistent in both positive and negative situations lending support to the third hypothesis. The stability of these processes across situations with distinct emotional valence further suggests that mindfulness processes generalize to a wide range of situations.

In support of the final hypothesis, findings from moderation analyses reveal that neither neuroticism nor extraversion significantly impacted the strength of the relationship between dispositional and situational use of mindfulness skills. Again, this pattern of findings was consistent across both positive and negative emotional contexts. Since neuroticism and extraversion are but two of several traits that, in theory, could alter the use of mindfulness skills in specific contexts, future investigations might consider other personality characteristics (e.g., hostility) that may affect the relationship between dispositional tendencies and situational behavior.

The total variance explained (*R* ^2^) in these models is relatively small. On one hand, this findings is encouraging because it suggests that while dispositional and situational mindfulness are related, this relationship is not sufficient to explain situational use of these skills. These findings should be interpreted with caution, however, because the measurement of situational variables itself can account for the observed variance. Previous studies have noted that most traditional techniques of psychological measurement, particularly self-report measures, rarely account for substantial variance in situational behavior [36]. Moreover, the measurement of situational use of mindfulness skills following structured imagery may be associated with common method variance (CMV), which might have contributed to the small variance accounted for within the statistical models. This possibility was not addressed in this investigation and may be an important consideration in subsequent research.

### Conclusions

The primary objectives of this investigation were to demonstrate that (1) situational measures of mindfulness reflects the same underlying constructs as dispositional measures of mindfulness and (2) that measures of dispositional mindfulness can predict situational use of mindfulness skills across a range of contexts, while accounting for the impact of personality traits.

Taken together, Studies 1 and 2 satisfy these objectives and indicate congruence between dispositional and situational measures. Study 1 suggests that attention awareness is deployed situationally at consistently high and low levels among those who report either high or low levels dispositionally. In contrast, mindful acceptance of emotions appears to depend on the nature of the situation in question. Moreover, whereas Study 2 demonstrates that dispositional assessment of mindfulness skills predicts use of these skills in both positive and negative situations, dispositional measures cannot fully explain situational behavior. These results further demonstrate that these particular dispositional measures (KIMS, AAQ) are accurate means for evaluating situational behavior despite their lack of direct measurement of at this level. Indeed, the current standard in the field of mindfulness research is dispositional measurement, which has been quickly expanding in recent years. Both the AAQ and KIMS are widely used, and although their dispositional nature is a conceptual limitation, our data suggest that they may reflect situational behavior.

This study carries implications for clinical practice and research. For instance, in estimating dispositional and situational use of mindfulness skills pre-treatment, clinicians may choose to utilize existing dispositional measures. However, it appears that a situational approach is useful in certain circumstances, including studies of treatment effectiveness. Mindfulness treatments may, for example, change one’s dispositional tendency toward entering mindful states without affecting situational behavior. Given that dispositional measurement may not fully characterize the use of these skills in clinically relevant situations, post-treatment situational assessment may be used to identify treatment-related change.

Importantly, recent work by Baer et al. [6] and Bach, Hayes, and Levin [37] indicate that other facets of mindfulness not directly addressed in this investigation, such as decentering, psychological flexibility, and cognitive defusion, operate as distinct processes. It is important to explore these additional components in order to establish their unique contribution to situational mindfulness skills, and to determine if they are distinct from broad tendencies toward dispositional mindfulness. As this investigation focused on one specific measure of mindfulness, it is limited in that such hypotheses could not be tested directly.

The present study suffers from several additional limitations. First, the cross-sectional design and exclusive reliance on self-report rating scales within an undergraduate population impact the potential to draw causal inferences. These issues also limit the generalizability of the current findings. Second, while situational ratings reflect the immediacy of attention awareness and emotion acceptance, imagery and self-report ratings did not occur simultaneously. As such, the use of spontaneous emotion regulation strategies may have immediate and unknown influences on self-report [38]. Related to this concern is the lack of standardization in the presentation of imaginal vignettes. Although all study participants were strongly encouraged to complete the questionnaire battery in a quiet place, the physical and/or psychological circumstances in which the participants responded to the survey may have influenced their ratings. Finally, these studies relied on imaginal exposure to present participants with emotionally evocative situations. While extant research supports the use of structured imagery as an analog to *in vivo* exposure for the induction of emotional reactions [39], meaningful differences in how acceptance and attention operate *in vivo*, as compared to imagery, may nonetheless impact self-report.

Future investigations should continue to refine techniques for ecological momentary assessment of mindfulness. Most notably, because situational ratings were not presented at the exact moment of structured imagery, developing a protocol that permits objective measurement of attention awareness and emotion acceptance would enhance this field considerably. Furthermore, the potential causal associations among mindful attention and acceptance should continue to be examined in prospective or longitudinal studies. In such studies, it would be important to examine how participant’s previous experience with mindfulness training impacts their behavioral tendencies. It is important also to discern how individuals who evidence high dispositional levels of mindfulness may differentially employ attention and acceptance skills. Such patterns of emotion regulation may be related to unique psychosocial outcomes such as the long-term improvement and reduction of psychological distress. Situational measures would likely contribute to predicting these outcomes and should be included in future studies.

Replication of these findings should be sought using other widely used measures of mindfulness (e.g., the MAAS,18). Also, replicating these findings using a clinical sample would be an important step in supporting attention awareness and emotion acceptance as features of psychosocial interventions. Paired with the results presented here, examination of the unique mechanisms in clinical populations could offer new options for assessment before, during, and after treatment.
